# Role of the La/K Compositional Ratio in the Properties of Waveguides Written by Fs-Laser Induced Element Redistribution in Phosphate-Based Glasses

**DOI:** 10.3390/ma13061275

**Published:** 2020-03-11

**Authors:** Pedro Moreno-Zarate, Francisco Muñoz, Belen Sotillo, Manuel Macias-Montero, Julia Atienzar, Marina Garcia-Pardo, Paloma Fernandez, Rosalia Serna, Javier Solis

**Affiliations:** 1 Electronic Engineering Department, Higher Technological Institute of Acatlan Osorio, Carretera Acatlan—San Juan Ixcaquistla k.m. 5.5, Acatlan de Osorio 74949, Mexico; 2 Institute of Ceramics and Glass (ICV, CSIC), Kelsen 5, 28049 Madrid, Spain; fmunoz@icv.csic.es; 3 Department of Materials Physics, Faculty of Physics, Complutense University of Madrid, 28040 Madrid, Spain; bsotillo@ucm.es (B.S.); arana@fis.ucm.es (P.F.); 4 Laser Processing Group, Institute of Optics (IO, CSIC), Serrano 121, 28006 Madrid, Spain; manuel.macias@csic.es (M.M.-M.); julia.atgar@gmail.com (J.A.); mgpardo9@gmail.com (M.G.-P.); rosalia.serna@csic.es (R.S.)

**Keywords:** fs-laser writing, photonic devices, glass, ion migration, element redistribution, waveguides, refractive index contrast

## Abstract

The local modification of the composition of glasses by high repetition femtosecond laser irradiation is an attractive method for producing photonic devices. Recently, the successful production of waveguides with a refractive index contrast (Δn) above 10^−2^ by fs-laser writing has been demonstrated in phosphate glasses containing La_2_O_3_ and K_2_O modifiers. This large index contrast has been related to a local enrichment in lanthanum in the light guiding region accompanied by a depletion in potassium. In this work, we have studied the influence of the initial glass composition on the performance of waveguides that are produced by fs-laser induced element redistribution (FLIER) in phosphate-based samples with different La and K concentrations. We have analyzed the contribution to the electronic polarizability of the different glass constituents based on refractive index measurements of the untreated samples, and used it to estimate the expected index contrast caused by the experimentally measured local compositional changes in laser written guiding structures. These estimated values have been compared to experimental ones that are derived from near field images of the guided modes with an excellent agreement. Therefore, we have developed a method to estimate before-hand the expected index contrast in fs-laser written waveguides via FLIER for a given glass composition. The obtained results stress the importance of considering the contribution to the polarizability of all the moving species when computing the expected refractive index changes that are caused by FLIER processes.

## 1. Introduction

Fs-laser writing of optical photonics components in dielectrics has been proven as a successful and versatile technique in many cases [1,2], although it is affected by some limitations, including the relatively small index contrast accessible in most cases (∆*n* = *n*_local_ − *n*_0_, typically well below 10^−2^), which is, in addition, strongly conditioned by the material itself [3]. In this context, the use of fs-laser induced element redistribution processes appears as a powerful alternative to produce high index contrast photonic devices [4]. Indeed, fs laser induced element redistribution (FLIER) processes have been studied long since. In the FLIER process, the bonds linking the network modifiers or the network formers are broken as a consequence of the laser energy deposition, and the elevated temperature induced, and the ions, including O^2−^, may diffuse, leading to a local modification of the material composition [5,6]. However, the production of real functional devices that are based on FLIER is relatively recent. FLIER has been successfully used to produce waveguides [7,8] and waveguide integrated optical amplifiers and lasers [9].

The most studied glasses for these FLIER based applications are phosphate-based glasses with La_2_O_3_ and K_2_O modifiers playing a key role in the production of light guiding structures. The guiding region is enriched in La^3+^ ions as a consequence of the laser energy deposition, leading to a substantial refractive index increase, reaching index contrast values ∆*n* > 10^−2^, while the K+ ions migrate in the opposite direction [7,8]. The origin of this index change due to fs-laser irradiation is very different than that traditionally observed in phosphate glasses with very low or no La_2_O_3_ content, where ∆*n* values typically in 10^−3^ range are observed due to the contraction of the phosphate network [10]. Such a kind of laser induced index modification in phosphate glasses is, in addition, very strongly dependent of the actual composition of the glass and on the laser processing conditions [10,11].

Although the FLIER process can be generally understood [12,13] as a form of Soret effect [14,15], the strong temperature gradients involved, the presence of convective flux, plasma and non-linear propagation effects, shockwaves, etc., add to the phenomenon a very large degree of complexity [4]. Indeed, the ultimate reason making different species migrate in opposite directions is far from being understood. Above a certain threshold, in the case of phosphates [7,8,16] and borates [17] that are modified with alkali and rare earth (RE) oxides, the glass former ions remain relatively stable in their positions, while alkali and RE move in opposite directions. In the case of silicates, the general trend is to observe the glass former ions to move to the hottest region of the interaction region, while modifiers move outwards to the colder one [6,13]. The need of preserving the local charge equilibrium upon ion migration in the laser affected zone suggests that the migration of one ion species requires the cross migration of one or more ions of a different element in the opposite direction. Therefore, the directional migration of a single type of ion alone looks improbable.

Whatever the detailed mechanisms underlying the process, we have shown that it can be controlled through the processing parameters [8,9] to the point of enabling the production of high-performance photonic components based on FLIER, as above indicated. However, the role of the initial composition of the glass on the final performance of the waveguides that are produced by FLIER has not yet been analyzed in detail. From the early work of Brow and coworkers on La-phosphate [18] and La-Al-phosphate [19] glasses, and the works on FLIER in La-K-phosphate glasses by Fernandez, Hoyo, Moreno-Zarate et al. [7,8,20], and in La-Na-borate glasses by Dias et al. [17], it is more or less clear that in the 0–10 mol·% range of La_2_O_3_ content in the glass, the local enrichment of a given region in La_2_O_3_ should give rise to a refractive index increase nearly proportional to the induced enrichment. At such low levels of La_2_O_3_ in the glass, La^3+^ ions will be fully coordinated by the terminal oxygens of the Q^n^ units that form the phosphate network [18], and the glass density will be directly proportional to the lanthanum addition. However, higher contents of La_2_O_3_ will produce a lack of enough terminal oxygens to coordinate all La^3+^, giving rise to formation of La-O-La bonds that will affect density and refractive index non-linearly. This index modification mechanism has also been confirmed in the case of a borate glass matrix, where, due to the different initial matrix density, the local index increase versus the local La^3+^ content is much stronger [17].

For the production of waveguides that are based on La_2_O_3_, La^3+^ ions cross-migrate K^+^ (or Na^+^) [7,8] ions to produce the higher index regions. While the enrichment of La_2_O_3_ in the guiding region favors the local index increase, the out-migration of K_2_O should tend to reduce it, to a much lower extent though, given the lower polarizability of K_2_O when compared to La_2_O_3_. As the local index finally achieved will be given by the polarizability contribution of all the oxides present [21], it is expected that the initial content of La_2_O_3_ as well as the initial La/K ratio in the matrix should play a strong role in defining the index contrast achievable upon laser-writing. Based on this idea in this work, we show how, by determining the contribution to the glass polarizability of the different glass constituents, it is possible to make accurate predictions of the final index contrast achievable by FLIER for a given initial composition. We demonstrate the feasibility of our approach for the case of waveguides produced by FLIER in La-K-phosphate glasses with different relative concentrations of La and K oxides.

## 2. Materials and Methods

### 2.1. Glass Samples Preparation, Composition, and Refractive Index Measurements

The phosphate glass samples that were used for fs-laser writing experiments were obtained through the melting and quenching of batches made by mixing K_2_CO_3_, La_2_O_3_, Al_2_O_3_, Er_2_O_3_, Yb_2_O_3,_ and (NH_4_)_2_HPO_4_ reagent grade powders, whose compositions are indicated in Table 1. The batches were slowly calcined up to 400 °C overnight in porcelain crucibles (79MF1a; Haldenwanger GmbH, Waldkraiburg, Germany), and then melted at 1450 °C during 4 h. The melts were then poured, crashed to very fine powder, and remixed in an agate ball mill (Pulverisette 6; Fritsch GmbH, Idar-Oberstein, Germany) for ten minutes in order to improve the homogeneity of the samples. The remixed powder was re-melted in Al_2_O_3_ crucibles that were coated with ZrO_2_ at 1450 °C to obtain the final glass samples, which were finally annealed above their glass transition temperature. This latter (T_g_) has been determined from the thermal expansion curves obtained in a 402 EP dilatometer (Netzsch-Gerätebau GmbH, Selb, Germany) at a heating rate of 5 K·min^−1^. The T_g_ of the three glass samples was comprised between 510 °C and 550 °C. The homogeneity of the samples, and the absence of striae and residual stress were tested by the shadow method and by means of a polariscope.

X-Ray Fluorescence (XRF) Spectroscopy was used to analyze the chemical composition of the final glasses in a MagicX 2400 spectrometer (Malvern Panalytical B.V., Eindhoven, The Netherlands) through the pearl method, using 0.3 g of glass with 5.5 g of Li_2_B_4_O_7_. Table 1 gathers the corresponding analyzed compositions in mol·%. The relative error in the determined compositions has been estimated to be within 2% with small differences, depending on the element considered. For the three samples, the loss of P during the glass production is very similar, leading to a P_2_O_5_ molar content nearly equal for the three glasses (~57–58 mol·%). There is, in addition, a strong incorporation of SiO_2_ from the crucible during the melting, which leads to approximately 15 mol·%.

After annealing, the samples were cut and polished to optical quality before optical characterization and fs-laser writing. The size of the produced samples was typically around 10 × 20 × 6 mm^3^. Their refractive index was characterized by means of spectroscopic ellipsometry in the near IR (800–1700 nm, including the S-L optical communications bands). Ellipsometric spectra were measured at several spatial locations in each sample in order to achieve statistically significant values. The acquired Ψ-spectra were fitted using the Cauchy equation for the refractive index *n* as a function of the wavelength, λ ($n\left(\lambda \right)=A+\frac{B}{\lambda^{2}})$. This dispersion equation describes very well the behavior of the refractive index of the glasses in the studied spectral region, where they are transparent (absorption coefficient k = 0). Table 1 presents the obtained coefficients A and B. Considering the uncertainties of the different parameters involved (angle positioning, wavelength, fit, ...), the absolute error in the obtained refractive index values has been estimated to be smaller than ±5 × 10^−3^ [20]. It must be considered though that, for the determination of the contribution of the polarizabilities of the glass constituents that are discussed in Section 3.1, the relevant parameter is the difference between the index of the samples with different composition, and such difference can be evaluated with accuracy close to 10^−3^ [22]. Indeed, in an ideal situation, by the direct inversion of the ellipsometric equations at a given wavelength without fitting the spectral dispersion, an index determination accuracy of close to 10^−4^ could be potentially achieved for sufficiently small instrumental errors [22].

### 2.2. Fs-Laser Writing and Characterization of the Light Guiding Structures

A femtosecond laser amplifier *Satsuma HP* (Amplitude, Pessac, France) operating at 1030 nm with a 500 kHz repetition rate and ~400 fs pulse width was used to produce light guiding structures in conditions that are similar to those reported by Del Hoyo et al. in Ref. [8]. The writing beam was circularly polarized and slit shaped (width 1.2 mm) before being focused in the sample 100 μm beneath the surface with a 0.68 NA aspheric lens. A motorized stage (x,y,z) allowed for scanning the sample transversely to the writing beam to generate the waveguides. We used pulse energies in the 600–750 nJ range and a sample scanning velocity of 60 μm/s, leading to structures with nearly constant diameter, as shown in the previous reference. Guiding structures that were typically 10 mm long were produced. After writing, the waveguide entrance and output facets of the samples were polished and optical transmission images of the structures were recorded by optical microscopy.

The performance of the waveguides was analyzed by coupling laser light in the waveguides at 976 nm or several wavelengths between 1450 and 1640 nm with an SMF-28 single-mode fiber. The inclusion of small amounts of Er_2_O_3_ and Yb_2_O_3_ in the composition of the sample was undertaken on purpose to facilitate the waveguides alignment during the characterization process, by means of the up-conversion green emission of the Er^3+^ ions upon excitation at 976 nm. After alignment, the output facet of the waveguide at the different wavelength was imaged with a 50× objective on an infrared camera to acquire the guided mode intensity distribution. Simulations of the measured mode field diameters (MFD), assuming a step-index waveguide, were then performed [23,24], leading to estimated Δn values that were consistent with the experimentally measured MFD values at the different wavelengths used (1440, 1490, 1534, 1590, and 1640 nm).

Finally, after modal characterization, one of the end facets of the waveguides was covered by sputtering deposition with a 7 nm-thick Au layer to avoid space charge effects during SEM imaging and EDX compositional analysis. These measurements were performed in a S440 Scanning Electron Microscope (SEM) (Leica Cambridge Ltd., Cambridge, UK) that was equipped with a Quantax X-ray microanalysis system (Bruker AXS, Karlsruhe, Germany) with a resolution of 125 eV.

## 3. Results and Discussion

### 3.1. Refractive Index and Molar Polarizabilities of the Samples

**Figure 1:**
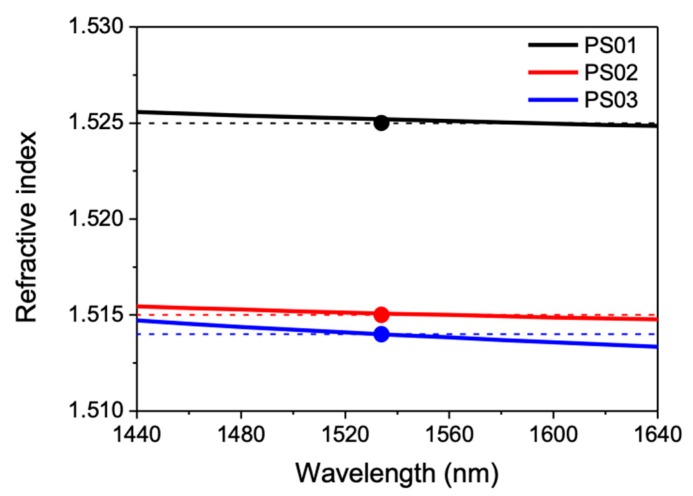
(Continuous lines) Refractive index of the samples PS01, PS02, and PS03 as a function of wavelength in the 1460–1625 nm interval determined from ellipsometry measurements. The symbols (and dashed lines) correspond to the index values of the three samples at 1530 nm estimated from the polarizabilities of the glass constituents, as explained in the text.

Figure 1 shows the refractive index of the three glass samples (PS01–PS03) as a function of wavelength in their transparency region (over the telecom bands S to L, 1460–1640 nm). It is worth noting that the index differences between the three samples can be clearly distinguished in the Cauchy fits of the Ψ-spectra. It can be seen that the spectral dependence shows a nearly parallel behavior for the different compositions with an increasing index for increasing La_2_O_3_ contents. Although the linear dependence of the index of phosphate-lanthanum glasses with the La_2_O_3_ content has been discussed in refs. [18,20], it must be noticed that, in this case, the comparison between the index of the three samples in terms of the La_2_O_3_ concentration alone is not fully consistent, since the concentration of K_2_O is very different in the three samples and all components contributed to the glass polarizability.

Further insight on the optical properties of these glasses and the origin of the index contrast in the laser processed samples can be achieved by analyzing their optical properties while using the polarizability approach, following the works from Dufy [21,25] and Dimitrov and coworkers [26]. From experimental refractivity measurements, by using the Lorentz–Lorenz relation, the molar electronic polarizability (*α_m_*) of a solid can be related to its refractive index (*n*) and molar volume (*V_m_*) [25]:

(1) $$\alpha_{m}=\frac{3}{4\pi }\frac{V_{m}}{N_{A}}\frac{n^{2}-1}{n^{2}+2}$$

For predominantly ionic media, the values of *α_m_* allow for the assignment of polarizabilities to individual cations and anions.

(2) $$\alpha_{m}=\sum \alpha_{i}+\sum \alpha_{anion\left(i\right)}^{2-}$$

where $\sum \alpha_{i}$ denotes the total molar cation polarizability and $\sum \alpha_{anion\left(i\right)}^{2-}$ the anions contribution. In general, while for most cations and anions their polarizabilities can be considered as nearly constant parameters that can be additively combined, in the case of oxide glasses, there is a much stronger variability of the oxide ion ($\alpha_{O}^{2-}$) polarizability that is related to the different polarizing power of the cations with which it is combined in the glass matrix. It is possible to estimate an average value for the polarizability of the oxide anion ($\alpha_{O}^{2-}$) by using an additive approximation, in which:

(3) $$\alpha_{O}^{2-}=[\left(\frac{V_{m}}{2.52}\right)\frac{\left(n_{0}^{2}–1\right)}{\left(n_{0}^{2}+2\right)}-\sum \alpha_{i}]{\left(N_{O}^{2-}\right)}^{-1}$$

where $\sum \alpha_{i}$ denotes the total molar cation polarizability and $N_{O}^{2-}$ the number of oxide anions [27].

In what follows, we use such an approximation to estimate the value of the oxide anion ($\alpha_{O}^{2-}$) polarizability for the P_2_O_5_ in the different samples, while using the cation polarizabilities included in Table 2 for the simple oxides, cations, and anions taken from Refs. [26,27,28,29]. For the case of Er_2_O_3_ and Yb_2_O_3_, although polarizability values that were derived from dielectric constant measurements up to MHz frequencies have been reported by Shannon [30] and for the ground state atoms of Er and Yb by Choi as the electric dipole polarizabilities by Choi et al. [31], due to the lack of reliable values for polarizabilities at optical frequencies we have used the ones that were reported for La_2_O_3_. This is a reasonable approximation, given that Er^3+^ and Yb^3+^ ions will experience a similar local environment as La^3+^ and show a similar electronic structure and ionic radii.

We have used the values of $\alpha_{cation}\left(A_{x}O_{y}\right)$ and those of ($\alpha_{O}^{2-}\left(A_{x}O_{y}\right))$ indicated in Table 2 in order to estimate the oxide anion polarizability in the P_2_O_5_ forming the glass samples ($\alpha_{O,\;}^{2-}\left(P_{2}O_{5}\right)$_glass_). The value estimated for ($\alpha_{O,\;}^{2-}\left(P_{2}O_{5}\right)$_glass_) (1.332 Å^3^, shown in parenthesis in Table 2) was obtained by minimizing the RMS difference between the electronic polarizability ($\alpha_{m}$) of the different samples, derived from refractive index values (Equation (1) that were obtained by ellipsometry), and the calculated values of $\alpha_{m}$ using [$\alpha_{O,\;}^{2-}\left(P_{2}O_{5}\right)$] as a fitting parameter. The validity of this approximation relies on the fact that P_2_O_5_ is the major constituent of the glass, and its concentration remains essentially unchanged in the synthesized samples. It is remarkable that the value that was obtained for ($\alpha_{O,\;}^{2-}\left(P_{2}O_{5}\right)$_glass_) is less than 1.5% smaller than the one corresponding to the single (pure) oxide and it is within the typical expected range for semi-covalent predominantly acidic oxides [28]. Therefore, the polarizability-based, estimated refractive index values show excellent agreement with the ellipsometry measurements for the three studied compositions, even with such minor change. The calculated values have also been included in Figure 1. Additionally, it must be emphasized, at this point, that, although the polarizability of La_2_O_3_ is about three times higher than that of K_2_O, the migration of K^+^ ions out from the guiding region might have a large impact in the local index contrast finally achieved, especially in those samples with large alkaline oxide content like PS03.

### 3.2. Morphology of the Structures, Guiding Performance and Refractive Index Contrast Estimations

**Figure 2:**
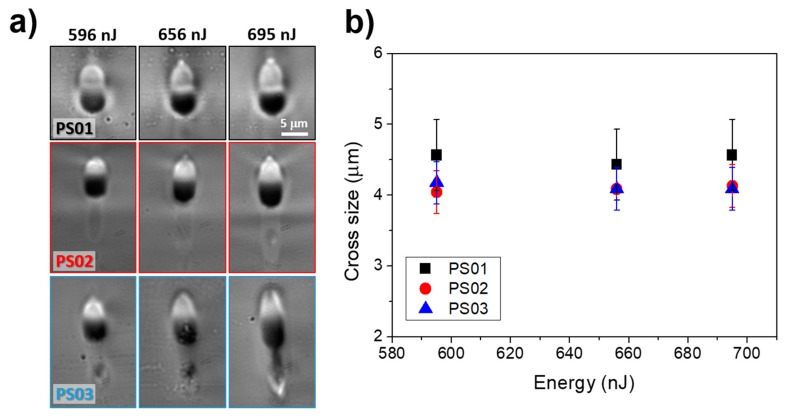
Optical microscopy images in transmission of structures written in the three different samples at different energies. The image plane corresponds to the output plane of the structures (transverse to the sample scan direction, laser incident from the top of the image) (**a**); Diameter of the refractive index increased region in the direction transverse to the laser beam propagation axis as function of the pulse energy (**b**) for the different samples.

Figure 2a shows a set of optical microscopy images of structures that are produced in the three samples for different pulse energies. The images correspond to a cross section view of one of the end facets of the laser written guiding structure. In all cases, a clear contrasted region that is closer to the surface on top of dark contrasted zone forms the structures. The local bright or dark contrast in the images is indicative of the local enrichment (or depletion) of La_2_O_3_ in the region causing an increase (or diminution) of refractive index, enabling the structures to guide light, as thoroughly discussed in [4,7,8]. Along with this main feature, we can see that the light guiding region adopts a symmetric, nearly-circular shape, whose transverse dimension (with respect to the beam incidence) remains nearly unchanged as a function of energy, as shown in Figure 2b. Still, as the energy increases, the longitudinal dimension of the La-enriched zone shows a somewhat stronger increase.

As above indicated, we have chosen the indicated pulse energies following Ref. [8] in order to be above the threshold for La-K cross migration and waveguide formation and below the limit where the heat accumulation effects turn out to excessive [32], leading either to a very strong increase of the size of the structures or strong damage [8]. The images in the figure also show that the length of the dark contrasted region increases with energy, which indicates that the region that is depleted in La_2_O_3_ gets larger, consistently with the slight increase in size of the light guiding zone. Furthermore, it is possible to appreciate for the three compositions that the vertical spread of the laser transformed region is longer than that of the strongly contrasted zones, above described. In all cases, the formation of a filament of increasing length (for increasing energy) can be appreciated, which is indicative for non-linear beam propagation effects (filamentation) [33,34].

In terms of morphology, for a given pulse energy, the most important difference between the three compositions analyzed is the apparent stronger contrast of the compositionally modified zones when the La_2_O_3_ increases, and the appearance of some internal stress in the form of light and dark grey stripes in the background of the structures in the PS01 sample (less visible in PS02). This feature could be indicative of a diminution of the thermal conductivity of the glass for increasing La_2_O_3_ contents. In this respect, it must be considered that, for sample PS03 (lowest La_2_O_3_ content), the expected larger thermal conductivity seems to modify the shape of the thermal gradient along the laser propagation axis during the process, which leads to the appearance of a second and weaker La^3+^ migration pole located at the bottom of the structure. This is clearly visible in the structure that was written at 695 nJ in PS03. This additional migration pole has been also observed in other glasses (borates) upon waveguide writing via FLIER [17].

**Figure 3:**
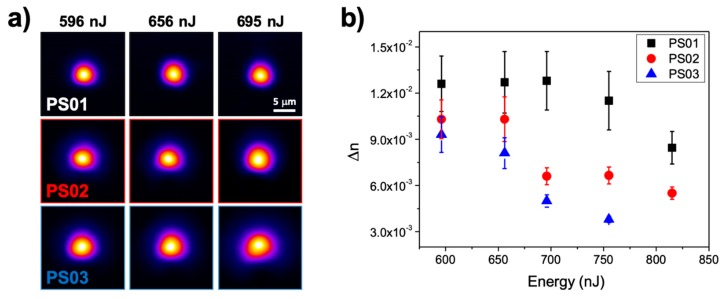
(**a**) Near field images of the guided modes propagated at 1534 nm by the structures shown in Figure 2a. The corresponding compositions and pulse energies are indicated in the (**b**) Refractive index contrast (∆*n*) determined from near field measurements (see text) as a function of the writing pulse energy for the three analyzed compositions. The values were estimated using images similar to those in (**a**) at six different wavelengths from 1420 nm to 1640 nm.

Figure 3a shows the near field images of light guided modes at 1534 nm corresponding to the structures in Figure 2a. For the three compositions, in the energy interval selected, the structures behave as single mode waveguides at this wavelength. It can be seen though that, for a given writing pulse energy, the mode field diameter decreases as a function of the initial La_2_O_3_ content of the glass sample, showing the minimum size for the PS01 sample. These features are consistent with refractive index contrast values (∆*n*) of at least 4−5 × 10^−3^, which increase with the initial La-content of the sample for a given pulse energy. A more precise determination of the index contrast can be made by using the mode field diameters that were measured at several wavelengths while using the method described in Refs. [23,24]. The error bars derive from the measured mode field diameters and the expressions that are included in those references. Figure 3b shows the corresponding values, where we have also included values that were obtained at higher writing energies to show the detrimental effect of excessive energy accumulation on the performance of the structures. For the lowest energy used, the index contrast already reaches values ∆*n* > 9 × 10^−3^ for all of the analyzed compositions, this value is higher the higher the initial La_2_O_3_ content of the sample. However, the behavior for higher pulse energies shows some clear differences for the three compositions under study. For sample PS01, the index contrast shows a slight increase with energies up to approximately 750 nJ, where ∆*n* starts to decrease. Sample PS02 shows a similar behavior, although the decay of ∆*n* starts at a lower pulse energy (~700 nJ). Such a decay can be understood in terms of a diminution of the local La_2_O_3_ concentration in the guiding region. This effect has been further confirmed by EDX measurements (see below), and it is easier to appreciate in the structure morphology as function of energy in sample PS03, where the contrast on the La-enriched zone starts to decrease with the pulse energy, even for energies as low as 650 nJ. The increase of the extension of the La-enriched guiding region (which gets less defined, see Figure 2a), along with the appearance of a second migration pole (see above), might explain the observed behavior. Remarkably, it is feasible to generate waveguides with an index contrast ∆*n*~10^−2^, even for this composition, with an initial La_2_O_3_ concentration of just 2.4 mol·%. To conclude this section regarding the guiding performance of the generated structures, it should be noticed that we have not made any attempt to optimize the propagation losses of the structures that range between 0.7 and 3 dB/cm, depending on the writing pulse energy and sample composition. Although we cannot discard that as a consequence of the FLIER process structural defects, like non-bridging oxygen hole centers (NBOHC’s) or dangling bonds, can be formed and contribute to optical losses, the propagation losses in waveguides produced by laser induced element redistribution are usually low (below 1 dB/cm), as shown for instance in Refs [7,8,9,17] of the manuscript. This is something that can be attributed to the thermal annealing driven by heat accumulation effects at high laser repetition rates [35].

### 3.3. Compositional Characterization and Calculation of the Expected Index Contrast Based on Polarizabilities

The composition of the laser modified regions was analyzed by EDX spectroscopy in a SEM in order to analyze the local refractive index variations quantitatively in terms of the compositional changes associated to the FLIER process. For such measurements, we used the waveguides showing larger index contrast difference for a given writing pulse energy (below damage threshold) among the different compositions (i.e., those structures written at 695 nJ, c.f. Figure 3b). The so-obtained local compositions were used to estimate the local refractive index while using the ionic polarizabilities that are discussed in Section 3.1 and compared to the refractive index contrast of the structures that were obtained from the mode imaging analysis.

**Figure 4:**
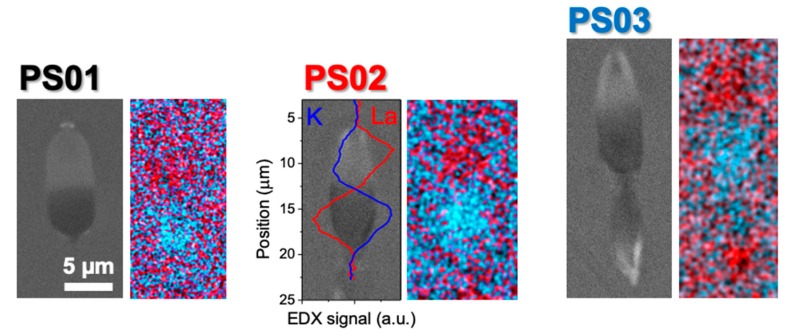
Scanning Electron Microscope (SEM) images and EDX maps of La (red) and K (blue) distribution in structures written at 695 nJ in samples (PS01, PS02, PS03) with different La_2_O_3_ and K_2_O content. The plot is a cross section of the distribution of both species along a vertical line passing through the center of the structure written in the PS02 sample. The scale bar is the same for all the images.

Figure 4 shows a set of SEM images of the waveguides in Figure 2 for a writing pulse energy of 695 nJ. EDX maps corresponding to the distribution of La and K in the laser affected zone are also included in the figure. The comparison of the SEM images with the corresponding optical microscopy ones in Figure 2 shows that the contrast of the regions enriched or depleted in La_2_O_3_ is similar in both techniques, as expected from previous works [7,8]. The La_2_O_3_ enrichment (or depletion) produces a positive (or negative) Z-contrast in the laser-modified regions, something that is seen in the transmission optical micrographs as a bright (or dark) contrast that is caused the local increase (or decrease) of refractive index. The EDX maps at the right of each SEM image allow for clearly appreciating the regions that are enriched or depleted in La_2_O_3_ and K_2_O.

For the pulse energy studied, only La and K show concentration changes above experimental error (~10% relative change with respect to the initial composition, depending on the element). It is important to consider that the X-ray emission efficiency associated to the L and M shells involved in the characteristic fluorescence of lanthanides is low, which makes it more difficult to quantify small changes in the local concentration of La in samples with initially low concentration of this element (e.g., PS03). Despite this, it is possible to appreciate the presence of the second La^3+^ migration pole located at the bottom of the structure written in sample PS03 in Figure 4. The plot overlying PS02 SEM image corresponds to the EDX signal that is associated to the Lα and Kα emission lines of La and K atoms, respectively, along a vertical line crossing the structure, evidencing the cross-migration of La and K species during the process. This line scan has been smoothed to show more clearly the overall distribution of La and K along the Z axis of the structure. Similar line scans with a spatial resolution of ~0.4 µm were performed in three different waveguides written at the same energy in each sample to determine the relative changes in the La^3+^ and K^+^ concentration in the guiding region. The concentration changes (relative to the pristine material) that were measured at the center of the guiding region in the line scans are included in Table 3, along with the corresponding error estimates. The later derive from the noise in the scan signal, and the variation of the measured values over the three waveguides analyzed. The so-determined compositional changes were used to calculate the local polarizability (and refractive index) at the center of the guiding zone of the written structures while using the values given in Table 1 and Equations (1) and (2). The corresponding index contrast values are also shown in Table 3 and compared to those determined from near field measurements of the light guided by the structures (cf. Figure 3b). The error of the ∆n values that are derived from local compositional measurements derives from the error in the local changes of La^3+^ and K^+^ concentration in the guiding region. The error in the determination of ∆n based on near field mode images is related to the experimental error in the measured mode field diameters and its influence in the expressions that are included in Refs. [23,24], as indicated in Section 3.2.

Table 3 clearly shows that, for the three samples, the index contrast that is estimated from the local compositional measurements at the center of the guiding region is consistent with the near field measurements of the guided modes. In the comparison, it must be noticed that the former is a local value, while the second is an average of the index change over the guiding region. It is also worth noting that, for the two samples with the higher La_2_O_3_ contents (PS01, PS02), the experimental error in the determination of the local K_2_O content is clearly smaller than in sample PS03. This is due to the peculiar morphology of the later, as can be seen in the optical images and SEM images of Figure 2 and Figure 4, which shows the presence of a secondary migration pole and a waist in the element distribution that also spreads over a much longer region. This makes the index contrast that is estimated by EDX measurements in sample PS03 to be well below that determined from mode field diameter measurements. In contrast, the agreement between both values is excellent for samples PS01 and PS02.

It is worth noting though that there is an apparent inconsistency in the values that are given in Table 3. At 695 nJ sample PS03 shows the highest relative enrichment in La_2_O_3_ in the guiding region (29%), while the highest index contrast is reached in sample PS01 with a lower La_2_O_3_ relative enrichment (25%). However, for sample PS03, such enrichment is equivalent to reach an absolute La_2_O_3_ local content of just 3.2 mol·%, while for sample PS01 the local La_2_O_3_ concentration reaches a value of 9%. In addition, in sample PS03, the local K_2_O concentration decreases from 17 mol·% to 15.3%, (10% change), while for sample PS01 (with a much lower initial K_2_O content) a relative decrease of 22% brings its local K2O content from 10.6 mol·% to 8.3 mol·%, an approximately similar diminution. In other words, an excessive depletion in K_2_O might be not compensated (in terms of index), unless its polarizability contribution is compensated with a sufficiently high La_2_O_3_ enrichment, something that in a sample with a low initial content of La_2_O_3_ would require a strong relative compositional increase.

**Figure 5:**
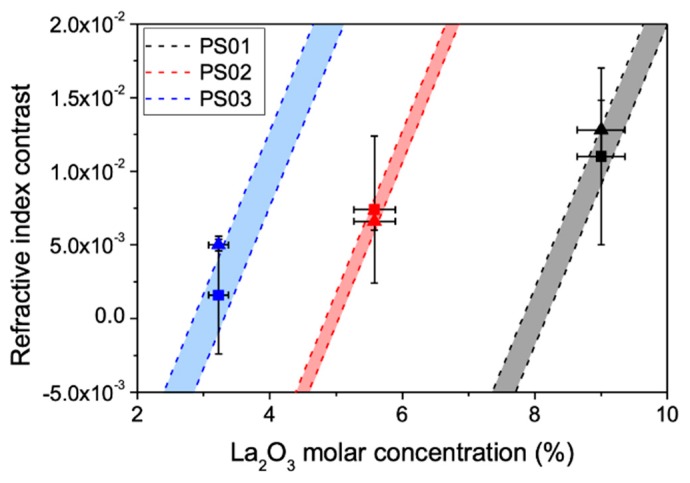
Refractive index contrast as a function of the local La_2_O_3_ concentration in the guiding region estimated from local compositional measurements (squares) and near field measurements (triangles). The shadowed regions mark the limits for the polarizability (composition) based estimates considering the maximum and minimum values of K_2_O content in the guiding region according to the error values that are given in Table 3.

In Figure 5 we have plotted the index contrast at the guiding region as a function of the molar concentration of La_2_O_3_ for the three different samples, including the values estimated from EDX measurements (square symbols) and those derived from near field measurements (triangle symbols). The error bars of the EDX-based values are directly related to the maximum and minimum values that the local K_2_O concentration can reach (c.f. Table 3). If we look at these error bars, we can see that, due to the relatively large amount of K_2_O in sample PS03 (17 mol·% c.f. Table 1), a relative K_2_O diminution in the guiding region of just 14% can lead to negative ∆*n* values, even for a La_2_O_3_ local enrichment of 30% (3.35 mol·%), while a depletion of 6% of K_2_O would lead to a positive contrast above 5 × 10^−3^ for the same La_2_O_3_ enrichment. The effect of the relative K_2_O depletion in reducing the index contrast in the guiding region is obviously reduced when the initial K_2_O content of the sample is smaller. Additionally, when the error in the K_2_O concentration is smaller, the consistency between polarizability-based index contrast estimations and near field measurements substantially increases, as it happens in samples PS01 and PS02. These features clearly show that we must consider the contribution to the local polarizability of all the moving species and not only of those generating the index increase in order to compute the index changes in the guiding region.

We have also included two dashed lines for each sample in Figure 5 to further illustrate the importance of the role of K_2_O in the local index of the structures formed by cross migration of La_2_O_3_ and K_2_O. They correspond to a calculation of the material polarizability while assuming a linear variation of the La_2_O_3_ for the maximum and minimum local concentration of K_2_O derived from the errors quoted in Table 3. Thus, the dashed lines provide a boundary for the expected index contrast of the three samples upon laser writing. The analyzed samples show K_2_O/La_2_O_3_ ratios from approximately 1 to 7 and La_2_O_3_ contents from ~2 to 7 mol·% and, in all cases, the predictions based on the polarizability approach are quite accurate. We can also use this approach to infer some features of the FLIER process occurring at lower writing pulse energies. In sample PS03, ∆*n* values that are close to 10^−2^ are experimentally observed at 596 nJ. A comparison of this value with the boundaries given by the shadowed region for this sample in Figure 5 suggests that the depletion of K_2_O in the guiding zone for this pulse energy should not be higher than ~6%, while the expected La_2_O_3_ enrichment should be close to 40%. This stronger La-enrichment and weaker K-depletion in the guiding region is consistent with both the initially lower La_2_O_3_ and the higher K_2_O content of the sample.

## 4. Conclusions

We have used ellipsometry measurements to compute the polarizability of the different glass constituents in phosphate-based glasses that were modified with different amounts of La_2_O_3_ and K_2_O, particularly the oxide ion ($\alpha_{O}^{2-}$) average polarizability in the P_2_O_5_ matrix with excellent results. The obtained values have been used to predict the refractive index contrast of optical waveguides that are produced by fs-laser induced element redistribution (FLIER) processes involving the cross migration of La^3+^ and K^+^ ions. The estimations were based on experimental EDX compositional measurements at the guiding region of structures that were produced in glasses with different La_2_O_3_ and K_2_O contents and they show excellent agreement with index contrast measurements based in near field imaging of the light guided modes. Along with the feasibility of predicting the expected index contrast in waveguides produced by FLIER in samples of different compositions, the results of the present manuscript evidence the importance of considering the contribution to the local polarizability of all the species that are involved in the FLIER process. The modelling results also justify the feasibility of producing waveguides with an index contrast close to 10^−2^ in samples with an initial La_2_O_3_ as low as 2 mol·%.

## Figures and Tables

**Table 1:** Molar composition (mol·%), and K_2_O/La_2_O_3_ molar compositional ratio of the samples as determined from XRF; and the corresponding Cauchy coefficients (A and B) for the refractive index as determined from the ellipsometric measurements.

Sample	K_2_O	La_2_O_3_	Al_2_O_3_	SiO_2_	P_2_O_5_	Er_2_O_3_	Yb_2_O_3_	K_2_O/La_2_O_3_	A	B
PS01	10.6	7.2	5.7	15.6	57.7	1	2.2	1.5	1.523	0.0006
PS02	14.4	4.5	4.9	14.6	58.3	1	2.2	3.2	1.514	0.0056
PS03	17.0	2.5	4.7	14.7	57.0	1.3	2.8	6.8	1.515	0.0069

**Table 2:** Polarizabilities of the different single oxides constituting the samples of Table 1. The cation and anion contributions are also indicated. The data, except for Yb_2_O_3_, where taken from Refs. [26,27,28,29]. The value indicated in parenthesis for P_2_O_5_ (bolded) has been calculated as indicated in the text.

Polarizability (Å^3^)	K_2_O	La_2_O_3_	Al_2_O_3_	SiO_2_	P_2_O_5_
Total	3.540	10.436	4.203	2.887	6.792
$\alpha_{cation}$	0.841	1.048	0.054	0.033	0.021
$\alpha_{O}^{2-}$	1.858	2.780	1.365	1.427	1.350 (1.332)

**Table 3:** Average local La_2_O_3_ enrichment and K_2_O depletion at the center of the guiding region of three waveguides written at 695 nJ in samples PS01, PS02 and PS03. The index contrast indicated (**∆***n* = *n*_local_ − *n*_0_) has been determined from the local composition of the guiding structures (“Estimated values”) using the values in Table 2 and Equations (1) and (2) or using the near field images of the modes propagated by the structures at several wavelengths (“Measured values”). The values shown were averaged over three waveguides written in the same conditions.

Sample	La_2_O_3_ Enrichment (%)	K_2_O Depletion (%)	Estimated ∆*n* (×10^−3^)	Measured ∆*n* (×10^−3^)
PS01	25 ± 5	22 ± 5	11 ± 6	12.8 ± 2.0
PS02	24 ± 7	9 ± 2	7.4 ± 5	6.6 ± 0.6
PS03	29 ± 6	10 ± 4	1.6 ± 4	5.0 ± 0.4
